# Narcissistic Leaders–Promise or Peril? The Patterns of Narcissistic Leaders’ Behaviors and Their Relation to Team Performance

**DOI:** 10.3389/fpsyg.2021.660452

**Published:** 2021-06-28

**Authors:** Ellen A. Schmid, Kristin Knipfer, Claudia V. Peus

**Affiliations:** ^1^Munich Business School, Munich, Germany; ^2^Technical University of Munich, Munich, Germany

**Keywords:** narcissism, leader narcissism, entrepreneurship, team performance, mixed methods, pre-founding teams, curvilinear relationships, qualitative interviews

## Abstract

Leader narcissism has attracted substantial attention in leadership research and organizational practice. Yet, the exact relationship between leader narcissism and performance remains unclear. In this paper, we set out to illuminate the narcissism-leadership-performance puzzle. We build on research that points to a curvilinear relationship between leader narcissism and performance and open the black box behind this curvilinear relationship. Thereby, we take into consideration the context, in which narcissistic leaders act, and explore their leadership behaviors in a compelling context: entrepreneurial teams. In a quantitative study, we found that a moderate level of leader narcissism was associated with the best team performance as assessed by the quality of a business plan. In a qualitative follow-up interview study, we explored the patterns of leadership behaviors shown by narcissists to better understand how different behaviors combine into effective versus destructive leadership, shaping team performance eventually. Finally, in an experimental online study using the scenario technique, we investigated the relevance of these leadership patterns associated with different levels of narcissism across contexts. The results of our multi-method and multi-source studies suggest that the most promising avenue to understand the narcissism-leadership-performance puzzle is that it depends on the levels of narcissism and more specifically that it depends on the patterns of behaviors narcissistic leaders show—the context seems to play a less important role.

## Introduction

There are many prominent examples of successful and visionary leaders who were labeled as ‘narcissists’ by the popular business press–think of Steve Jobs, Bill Gates, or Larry Ellison. While these leaders have been praised for their passion, vision, and innovation capacities (Sharma and Grant, 2011; Shah and Mulla, 2013), they have also been criticized for poor treatment of employees, lack of empathy, and arrogance (Maccoby, 2000; Gladwell, 2002). This dialectic view of narcissism, encompassing both positive as well as negative aspects, has received substantial attention in leadership research, yet it also poses one of the biggest puzzles that remains until today since “research has not produced consensus concerning whether narcissistic leaders hinder or benefit their organizations” (Grijalva et al., 2015, p. 1).

Different explanations have been proposed for this ‘narcissism-leadership-performance puzzle.’ Recent research has introduced promising new theories into the narcissism leadership landscape: For example, Liu et al. (2017) used trait-activation-theory to explain the relation between leader narcissism and self-interested behaviors, others argue that the sub-dimensions of narcissism need to be taken into consideration (Macenczak et al., 2016; Helfrich and Dietl, 2019). Yet, the two most promising avenues to explain the narcissism-leadership-performance puzzle are that whether narcissism in leaders is helping or hindering performance depends on (1) the *level of narcissism* (Grijalva et al., 2015) and (2) the *context* (Braun, 2017).

First, in a recent meta-analysis, Grijalva et al. (2015) found that narcissistic leadership and perceptions of leader effectiveness are curvilinearly related. Thus, it seems that a medium level of narcissism is conducive to performance, whereas high levels are not. While this has given us a much clearer understanding of the narcissism-leadership-performance puzzle, the question what causes this curvilinear relationship remains unanswered. We expand on findings Grijalva et al. (2015) and set out to answer the ‘why?’ and to open the ‘black box’ behind the curvilinear relationship of leader narcissism and performance. To this end, and as the nature and degree of narcissism is reflected in leaders’ behavior (Braun, 2017), we argue that the answer will be found in the actual behaviors that leaders with different levels of narcissism show. Hence, we explored the *patterns of leadership behaviors* shown by narcissistic leaders to better understand how different behaviors combine into effective versus destructive leadership, shaping performance eventually.

Second, another compelling explanation for the narcissism-leadership-performance puzzle is that it depends on the context whether a narcissistic leader will drive or derail team performance (Braun, 2017). Accordingly, we deem it important to additionally consider the context in which narcissistic leaders act (see also Nevicka et al., 2013). Concretely, narcissistic leader behaviors should be effective in contexts that gives them ‘a stage to shine’ (Nevicka et al., 2011b). We argue that entrepreneurship is a context, where leader narcissism may unfold positive effects (Engelen et al., 2016; Reid et al., 2018): It is a context that involves high ambiguity, uncertainty, and need for orientation (Knipfer et al., 2018); this calls for a leader who creates a strong vision and convinces other team members to follow this vision (Baum et al., 1998; Cooney, 2005). At the same time, narcissists are drawn to entrepreneurship and likely emerge as leaders of entrepreneurial teams (Mathieu and St-Jean, 2013; O’Reilly et al., 2013; Baldegger et al., 2017). Hence, entrepreneurial teams are a particularly suitable context to study the effects of leader narcissism (Hogan and Kaiser, 2005; Reid et al., 2018).

In summary, this research aims to illuminate the narcissism-leadership-performance puzzle. We build on research that points to a curvilinear relationship between leader narcissism and performance and open the black box behind this curvilinear relationship to provide evidence on which patterns of leadership behavior would drive or derail team performance. Moreover, we take into consideration the context, in which narcissistic leaders act, and explore their leadership behaviors in a compelling context: entrepreneurship. Answering a recent call for more research on narcissism in entrepreneurship, we are, to the best of our knowledge, the first to show a curvilinear relationship between leader narcissism and performance of entrepreneurial teams. Finally, we investigate the relevance of patterns of leadership behaviors associated with different levels of narcissism across contexts. Overall, we bring together two streams of research that have offered explanations for the narcissism-leadership-performance puzzle.

## The Narcissism-Leadership-Performance Puzzle

The concept of narcissism goes back to the Greek myth of Narcissus, the young man who fell in love with his own image. Based on this ancient tale, Freud (1914) introduced the concept of narcissism. Nowadays, it is defined by the American Psychological Association (Miller et al., 2010) as a set of behaviors described by predominant patterns of grandiosity along with a need for admiration and lack of empathy. Narcissists have been characterized by a sense of personal superiority (Campbell et al., 2004), grandiosity (Morf and Rhodewalt, 2001), and dominance, as well as by being assertive (Judge et al., 2006), desiring power (Emmons, 1987), and longing for attention and confirmation of their superiority (Bogart et al., 2004). Furthermore, narcissists have been described as lacking true empathy and therefore being exploitative, taking credit for others’ accomplishments, and shifting blame to others (Brunell et al., 2008; Rauthmann, 2012). They demand unquestioning devotion and loyalty from followers (Harwood, 2003; Rosenthal and Pittinsky, 2006). Narcissistic leaders are more self-serving than their humbler counterparts and tend to allocate scarce organizational resources to themselves (Van Dijk and De Cremer, 2006). When confronted with criticism or negative feedback, they frequently react with aggressive and hostile behaviors (Exline et al., 2004), and when they perceive to be treated unfairly, they are likely to respond with self-interested behaviors (Liu et al., 2017).

However, the first impression narcissists make on others stands in sharp contrast to the negative descriptions summarized above. In fact, narcissists’ self-enhancement usually appeals to other people when first meeting them (Paulhus, 1998; Brunell et al., 2008). Narcissists are perceived as interesting, charming, and interpersonally skilled (Deluga, 1997; Chatterjee and Hambrick, 2007). They are seen as confident and extraverted (Emmons, 1987; Campbell et al., 2004; Judge et al., 2006) and thereby portray the image of a prototypical effective leader (Emmons, 1987; Paulhus, 1998; Taylor et al., 2003; Campbell et al., 2004; Judge et al., 2006). This is one reason why narcissists are likely to be chosen as leaders (Judge et al., 2006; Brunell et al., 2008; Nevicka et al., 2011b), a fact that may in turn explain the seemingly high number of narcissists in CEO positions.

Not surprisingly, considering the two sides of narcissists, research to date suggests that a narcissistic leader can have positive as well as negative effects on team performance (for a review, see Braun, 2017). To provide a short synthesis that does not claim completeness: Leader narcissism relates to a broad range of negative follower outcomes such as dissatisfaction with the leader, counterproductive workplace behavior, or disengagement from work (Judge et al., 2006; Amernic and Craig, 2010; Godkin and Allcorn, 2011; Graham and Cooper, 2013; Rijsenbilt and Commandeur, 2013; Grijalva and Newman, 2015; Braun et al., 2018; Wirtz and Rigotti, 2020)–all of which should be detrimental for performance. In fact, Ham et al. (2018) found a negative relationship between CEO narcissism and firm profitability. In contrast, narcissistic leaders are prone to initiate mergers and acquisitions (Aktas et al., 2016), to engage in internationalization strategies (Oesterle et al., 2016), and to invest in new technology (Gerstner et al., 2013)–all of which may contribute to performance. For instance, Reina et al. (2014) found that CEO narcissism is positively related to firm performance, and Olsen et al. (2014) found that CEO narcissism positively relates to a firm’s earnings per share and stock price. Finally, Chatterjee and Hambrick (2007) found leader narcissism to be positively related to strategic dynamism, that is, the degree to which a strategy adapts to changing environments.

The above-mentioned research illustrates the well know narcissism-leadership-performance puzzle that is summarized by the question “Is it good or bad for a leader to be a narcissist?” (Rosenthal and Pittinsky, 2006, p. 619). To solve this puzzle, different explanations have been proposed. We argue that the two avenues that are most promising are that it depends on the *level of narcissism* (Grijalva et al., 2015) and the *context* (Braun, 2017).

### The Level of Narcissism Leverages Performance

An item response analysis of the Narcissistic Personality Inventory (Ackerman et al., 2012) revealed that individuals with high levels of narcissism tend to confirm destructive aspects (e.g., arrogance and exploitativeness), whereas individuals with medium levels of narcissism confirm constructive aspects of narcissism (e.g., confidence and assertiveness). This suggests that leaders with a moderate level of narcissism show productive behaviors–which will be beneficial for performance. In support for this argument, a meta- analysis of leader narcissism (Grijalva et al., 2015) found evidence for a curvilinear relationship between leader narcissism and perceptions of leader effectiveness, suggesting that a medium level of narcissism is optimal, whereas both very low and very high levels are detrimental for leader effectiveness. In a recent study, Uppal (2020) were the first to show a curvilinear relationship between CEO narcissism and objective measures of firm performance.

Whereas the meta-analysis by Grijalva et al. (2015) provides strong evidence for a curvilinear relationship between leader narcissism and leader effectiveness, we still do not understand what causes this curvilinear relationship. We argue that the key to understanding the dualism of leader narcissism may lie in the concrete behaviors that leaders with different levels of narcissism show (Braun, 2017) and how they combine into effective or destructive patterns of leadership behaviors.

### The Context Shapes the Effects of Narcissism on Performance

Previous research pinpoints the relevance of the context for the study of narcissism and its outcomes. Braun (2017) concludes from her review of the literature, that “narcissistic leaders might be more ‘fit for purpose’ in some environments than in others.” More concretely, she argues that narcissistic leaders might be successful in situations that involve high levels of uncertainty or under dynamic market conditions that require risk-taking and bold decision-making. A context in which narcissism may indeed be beneficial is entrepreneurship as, there, transformative innovations and bold decisions are needed (Baum et al., 1998). We thus argue that entrepreneurship is a context that gives narcissistic leaders a stage to shine.

Entrepreneurship is defined as the “processes of discovery, evaluation, and exploitation of opportunities” (Shane and Venkataraman, 2000, p. 218). It is a context that is characterized by unclear outcomes and high ambiguity, and is as such a *weak situation* (Shamir and Howell, 1999). In weak situations, people rely on someone who has a clear vision and is a powerful team leader, someone who can reassure them with their self-confidence (Maccoby, 2000; Padilla et al., 2007; Campbell and Campbell, 2009). In line with this, previous research on entrepreneurial teams has pointed toward the significance of the leader for the success of the entrepreneurial endeavor (Vecchio, 2003; Bryant, 2004; Cogliser and Brigham, 2004; Ensley et al., 2006; Knipfer et al., 2018). Leadership is therefore an area of research within entrepreneurship that has seen much focus over the last decade and that warrants further study (Reid et al., 2018). This is especially true for nascent entrepreneurial teams in the pre-founding phase. While this phase is crucial for new venture creation, it is at the same time very different from later stages of the entrepreneurial endeavor (Foo et al., 2006; Bergmann and Stephan, 2013). In this phase, the teams need to recognize business opportunities, select the most promising ideas, and continuously refine their business model (Ardichvili et al., 2003). Pre-founding entrepreneurial teams operate within a dynamic and uncertain context (McMullen and Shepherd, 2006), typically lack well-defined goals, structures, and work processes (Foo et al., 2006), and face the challenge of developing a shared vision and team goals for their entrepreneurial endeavor (Vyakarnam et al., 1999; Fernald et al., 2005; Ensley et al., 2006).

First empirical evidence points to the fact that charismatic leadership can be particularly effective in the pre-founding phase, as this type of leadership seems to help teams develop their business from a vague idea into a tangible business plan, and, at the same time, establish effective teamwork (Ensley et al., 2006). Underlying this relation are leader behaviors such as developing a clear vision and communicating it in a way that inspires followers (Knipfer et al., 2018). These are behaviors narcissists have shown to be good at Galvin et al. (2010). In fact, narcissism was identified as an important area of leadership research in entrepreneurship in a recent review, although one that is still under-researched. With our research, we set out to answer this call for more research on narcissism in entrepreneurship by Reid et al. (2018).

It seems that narcissists are drawn to entrepreneurship as a context where they can shine as entrepreneurial leaders (Mathieu and St-Jean, 2013; O’Reilly et al., 2013; Baldegger et al., 2017), and narcissism was related to entrepreneurial intention (Wu et al., 2019). In fact, earlier research suggests that narcissists are more likely to be founders of surviving firms than those lower on narcissism (O’Reilly et al., 2013), and that narcissistic CEOs increase entrepreneurial orientation within their organization. Yet, CEO narcissism was also associated with increased instability in firm performance (Wales et al., 2013). Buttice and Rovelli (2020) found that leader narcissism relates negatively to success on crowdfunding sites, and Liu et al., 2019 found that narcissism makes it less likely for entrepreneurs to learn from failure.

It seems that the narcissism-leadership-performance puzzle is also present in the entrepreneurship context, and we argue that it will also depend on the level of narcissism whether a narcissistic leader will be successful or not in the context of entrepreneurship. However, so far research has not explored this assumption.

## Hypotheses

Grijalva et al. (2015) provided strong evidence for a curvilinear effect of narcissism on perceptions of leader effectiveness, suggesting that a medium level of narcissism is optimal whereas both very low and very high levels are detrimental. We build upon their research and, based on their argumentation, we propose a curvilinear relationship between narcissistic leadership and performance also at the early stage of an entrepreneurial venture. Specifically, we assume that low levels of narcissism will not be beneficial for entrepreneurial team performance because the entrepreneurial context, especially at the early stage, calls for a strong team leader who is confident and ready to make bold decisions. However, the positive effect of narcissistic tendencies of an entrepreneur may turn into “a curse” (Grijalva and Harms, 2014, p. 121). Overconfident entrepreneurial leaders may be self-defeating in the long run as those entrepreneurs are likely to ignore signals of difficulties and failure (Hayward et al., 2006). Moreover, we assume that high levels of narcissism expressed in low empathy, a tendency to exploit others, a sense of entitlement, arrogance, and aggression are detrimental for entrepreneurial team performance. Instead, we propose that a medium level of narcissism will be most beneficial in the entrepreneurship context because it represents the effective characteristics of narcissism such as confidence and assertiveness.

***Hypothesis 1a:*** *The relationship between leader narcissism and entrepreneurial team performance is curvilinear.*

***Hypothesis 1b:*** *Entrepreneurial team performance is highest when leader narcissism is at a medium level and lowest at very low and very high levels of narcissism.*

Moreover, we aim to uncover the patterns of leadership behaviors underlying this relationship and assume that medium levels of narcissism are associated with constructive entrepreneurial leadership behaviors such as showing confidence and empowering others, whereas very low and high levels of narcissism are likely to be related to destructive entrepreneurial leadership behaviors such as timidity or dominance.

***Hypothesis 2:*** *Medium levels of leader narcissism are associated with constructive leadership behaviors, whereas low and high levels of leader narcissism manifest in destructive leadership behaviors.*

To test our hypotheses, we conducted a field investigation of nascent entrepreneurial teams using a multi-method and multi-source approach: In Study 1, we tested the hypothesis that the relationship between leader narcissism and team performance is curvilinear using a survey to assess followers’ perceptions of narcissistic leadership and expert ratings of the quality of business planning. In Study 2, we examined leadership behaviors in more detail to shed light on the proposed curvilinear effect of leader narcissism and team performance. As quantitative methods would be insufficient to capture the nuances and meanings of leadership behaviors, we chose a qualitative approach using qualitative interviews with both the leaders and members of the entrepreneurial team they lead.

Additionally, in a third study, we set out to investigate if the patterns we found in Study 2 are in fact specific to the entrepreneurship context or if they also apply in other, more traditional contexts, such as large corporations. Hence, in Study 3, we examined the effectiveness of the leadership patterns associated with low, medium, and high levels of narcissism across two contexts (i.e., entrepreneurship and corporate).

## Study 1: Curvilinear Effect of Leader Narcissism on Entrepreneurial Team Performance

### Sample and Procedure

We gathered data in collaboration with an innovation and start-up center in Germany. We studied entrepreneurial teams who participated in a 4-month venture creation program that combines education and incubation (Lackéus and Williams Middleton, 2015). After 4 months of weekly training and teamwork, the teams submitted a business plan, which concluded the program. Before the evaluation of the business plan were made available, we administered a survey to 252 participants. The participants were asked to sign a consent form in which they granted access to their performance evaluations. The final sample included 140 members of 58 teams of three to five team members (41.4% with three team members, 53.4% with four team members). Since the start-up center works closely with a local university, many of the participants had ties to the university, either being students, alumni, doctoral candidates, or researchers. Participants were on average 23.6 years old (*SD* = 3.3). 21.4% of the participants were female and 15.7% were international participants.

### Measures

#### Team Perception of Leader Narcissism

Every member of the team rated the leader based on seven forced-choice items from the Narcissistic Personality Inventory (Ames et al., 2006). The German version was taken from Schütz et al. (2004). A sample item is “He/she thinks, he/she is a special person” versus “He/she thinks he/she is no better or worse than other people.” To increase reliability, the final narcissism score was calculated based on six items (α = 0.64). Although the reliability of our narcissism measure was modest, it is acceptable for a forced-choice item scale (e.g., Jackson et al., 1973) and also comparable to the reliability reported in previous research (e.g., Ames et al., 2006; Goncalo et al., 2010; Iliescu et al., 2015). To test for the validity of our shortened scale, we collected additional data from 35 team members in the same program using the validated 16-item measure of the NPI. The correlation between the 16-item and our shortened 6-item measures was *r* = 0.796, indicating that the shortened measure is adequate to assess leader narcissism.

Prior to aggregation of leader narcissism to a team-level variable, we calculated *r*_*wg*_, *ICC(1)*, and *ICC(2)* as indicators for within-team agreement and between-team variance and tested for significance of the *ICC(1)* values. For team perceptions of leader narcissism, the average *r*_*wg*_ was .0.97, which indicates high within-team agreement (Bliese, 2000). The *ICC(1)* was 0.27 (*p* < 0.01), indicating a medium to large team effect (Bliese et al., 2002), and the *ICC(2)* was 0.47. Given the rather small team size, these values are acceptable and imply that the aggregation of individual ratings to a team-level variable was adequate and justified (Klein and Kozlowski, 2000). The high within-team agreement indicates that the individual perceptions of the leader’s narcissism were consistent, giving us further confidence that the aggregated measure of narcissism is an adequate representation of leader narcissism.

#### Team Performance

Previous research shows that the quality of the business plan is a good predictor of a new venture’s success at later phases (Shepherd et al., 2003; Foo et al., 2006; Frese et al., 2007). Hence, we used the quality of business planning as a performance measure on the team-level. To ensure validity of our performance measure, we used a three step assessment approach: First, the written business plans were rated by two entrepreneurship experts using a Likert-scale ranging from 1.0 (*very poor*) to 10.0 (*outstanding*). This assessment was based on predefined evaluation criteria. The first expert rated all business plans, and the second entrepreneurship expert evaluated one third of the business plans. The experts’ judgments were significantly correlated, *r* = 0.79, *p* < 0.001. Inter-rater reliability computed as intra-class correlation was 0.77, *p* < 0.001, indicating strong agreement (LeBreton and Senter, 2008). Third, to further validate the quality of the business plan as an outcome measure, we hand-collected information about the teams using archival data from the innovation center as well as from the Internet. Of 23 teams, whose business plans were evaluated very positively (i.e., between 9.0 and 10.0), seven were granted access to advanced training and coaching sessions, nine won awards in business competitions and/or received funding, and three pursued activities to start a business. In contrast, of the 35 teams whose business plans were evaluated as 8.0 or lower, only one was granted access to advanced training and coaching sessions, only one was awarded/received funding, and two pursued activities to start a business.

#### Control Variables

We carefully considered variables that may confound the proposed relationships (Spector and Brannick, 2011; Bernerth and Aguinis, 2016). We included team size as a control variable because it was shown to influence team performance (Jin et al., 2016). We also ascertained age and gender of the lead entrepreneur because both can leverage the effects of leadership (Kearney, 2008) by serving as “diffuse status cues” in the legitimization of influence attempts (Jung et al., 2015).

### Results

Table 1 shows the means, standard deviations, and interrelations of the study variables on the team-level. In Hypothesis 1, we postulated that the relationship between leader narcissism and team performance is curvilinear such that medium levels of narcissism are associated with higher performance, whereas low and high levels of narcissism relate to lower performance. We used hierarchical ordinary least-squares (OLS) regression analyses to test this hypothesis (see Table 2). We entered our control variables, namely, age and gender of the entrepreneurial leader as well as team size in Step 1. In Step 2, we entered the linear term of team perceptions of leader narcissism, and in Step 3, we entered the squared term of team perceptions of leader narcissism to test whether the squared term explains significant variance in the dependent variable team performance above and beyond the linear term of leader narcissism, which would provide evidence for the proposed curvilinear relationship (Cohen et al., 2002).

**TABLE 1:** Descriptives and intercorrelations of study variables (Study 1).

Study variable	1	2	3	4	5
1. Leader narcissism					
2. Team performance	−0.225°				
3. Leader age	–0.115	–0.038			
4. Leader gender	0.223°	–0.086	0.186		
5. Team size	–0.125	–0.126	0.095	–0.159	
*Mean*	*0*.*24*	*8*.*19*	*24.25*	*NA*	*3*.*64*
*SD*	*0*.*21*	*0*.*24*	*3*.*08*	*NA*	*0*.*58*

N = 58 teams. °*p* < 0.10; *p < 0.05; **p < 0.01; ***p < 0.001.

**TABLE 2:** Hierarchical regression analysis (Study 1).

Model	Variable	*B*	*SE*	*t*
1	(*Constant*)		1.72	3.86
	Team size	0.13	0.29	0.92
	Leader gender	0.10	0.40	0.70
	Leader age	0.07	0.06	0.05
2	(*Constant*)		1.72	4.56
	Team size	0.12	0.28	0.87
	Leader gender	0.16	0.40	1.11
	Leader age	–0.03	0.06	–0.22
	Leader narcissism	–0.24	0.83	−1.70°
3	(*Constant*)		1.69	3.79
	Team size	0.08	0.28	0.57
	Leader gender	0.16	0.39	1.16
	Leader age	0.02	0.05	0.13
	Leader narcissism	0.70	2.62	1.56
	Leader narcissism^2^	–0.99	3.40	−2.21*

R^2^ = 0.16, Δ*R*^2^ = 0.08, *F*(1,49) = 4.86, *p* < 0.05. *N* = 58 teams. °*p* < 0.10; *p < 0.05.

Leader narcissism showed a marginally significant negative relation with entrepreneurial team performance (β = −0.241, *p* < 0.10). When entering thee quadratic term, the linear term was not significant anymore (β = 0.702, *ns*), providing the basis for our more nuanced predictions of a curvilinear relationship of team perceptions of leader narcissism and team performance. In fact, the quadratic term proved to be significant and explained variance in addition to the linear term for team performance (β = −0.987, *p* < 0.05). The inclusion of the quadratic term of team perceptions of leader narcissism into the regression equation resulted in an improvement of the *R*^2^ from 0.077 to 0.160, *F*(1,49) = 4.861, *p* < 0.05.

**FIGURE 1:**
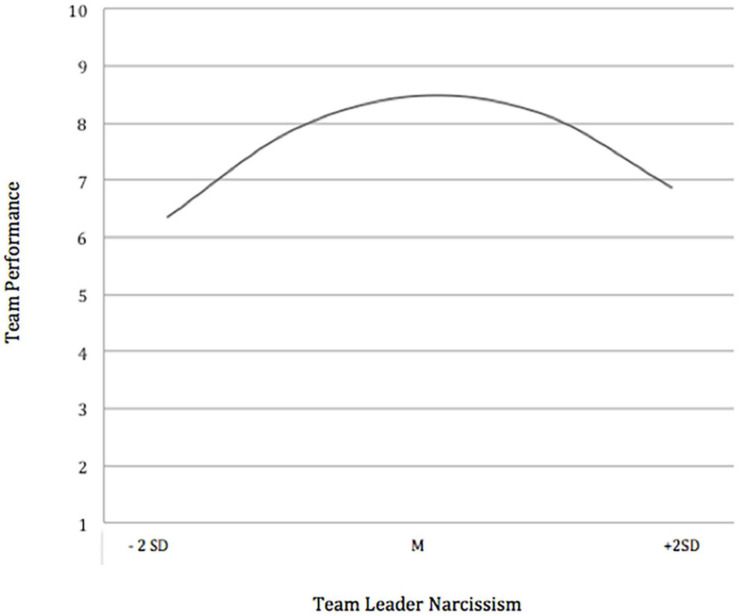
Curvilinear effect of team leader narcissism on entrepreneurial team performance (Study 1). To aid interpretation, team performance values were calculated using regression coefficients of a hierarchical regression analysis without controls and non-standardized values for leader narcissism (*y* = 7.99 + 3.63*x – 6.82*x^2^).

To visually explore the curvilinear relationship, we calculated the outcome variable team performance by inputting different values for the team perceptions of leader narcissism (the mean, two standard deviations below the mean to two standard deviations above the mean). As predicted, the results showed an inverted U-shaped relationship between team perceptions of leader narcissism and team performance (see Figure 1). Thus, Hypothesis 1 was fully supported.

## Study 2: An Exploration of the Patterns of Narcissists’ Leadership Behaviors

Study 1 showed a curvilinear relationship between leader narcissism and entrepreneurial team performance. Yet, the processes driving this relation remain unclear. The aim of Study 2 was to explore which specific leadership behaviors underlie the curvilinear effect we found in Study 1. Based on Ackerman et al. (2012) and Grijalva et al. (2015), we assumed that a medium level of leader narcissism is associated with effective behaviors, whereas a high level of leader narcissism should be related to destructive behaviors. We further hypothesized that a very low level of leader narcissism is also detrimental for team performance.

Thus, Study 2 sought to be particularly sensitive to the different behavioral patterns that leaders with low, medium, and high levels of narcissism showed toward their team and how these behavioral patterns impacted team performance eventually.

### Sample and Procedure

At the end of the venture creation program, we invited team leaders and team members to discuss their teamwork in individual semi-structured interviews of 45 to 60 min. Participation was voluntary. All interviews were conducted in person, and either in German or English. Participants were asked to describe how the team worked together during the past months and to elaborate on challenges that occurred. Then they were asked to describe the behavior of the team leader, i.e., team members were asked to describe how they experienced the team leader’s behavior, whereas team leaders were asked how they behaved. 25 interviews were conducted and the final sample included 13 entrepreneurial leaders and 12 team members. With this, 18 teams form the 58 teams from Study 1 were represented in the interviews.

We used a combination of deductive and inductive analysis techniques to identify relevant leadership behaviors (see Gardner, 2012). This combination was used because leader narcissism is well described in the literature (therefore a deductive approach was used) but, at the same time, we wanted to ensure that relevant behaviors that emerged from the data were not omitted (therefore an inductive approach was used).

### Development of Codes

In order to generate codes that are unbiased, we followed best practice recommendations (Strauss and Corbin, 1990) and applied four steps: In a first step, two of the authors read and re-read the interview transcripts and compiled interview-specific descriptive summaries individually (Strauss and Corbin, 1990; Foldy et al., 2008; Ospina and Foldy, 2010) in order to document emerging major themes and preliminary conclusions about the leadership behaviors of the team leader.

In a second step, the authors compared major themes that they identified to detect similarities and differences with regard to leadership behaviors across all interviews, and assigned descriptive codes for the emerging themes after discussing them intensely (Strauss and Corbin, 1990; King, 1994). These descriptive codes were partly based on the well-established items of the NPI scale provided by Ackerman et al. (2012). To ensure an unbiased approach, we also referred to the response frequencies that Ackerman et al. (2012) had identified: For codes that characterized medium levels of leader narcissism, we referred to NPI items for which Ackerman et al. (2012) reported response frequencies >0.50. Ackerman et al. (2012) argue that those items describe moderate levels of leader narcissism rather than extreme behaviors (e.g., “I like to take responsibility for making decisions,” proportion endorsed was 0.57, i.e., 57% of the participants agreed with this item). To develop codes that represented high levels of leader narcissism, we referred to items for which Ackerman et al. (2012) reported response frequencies that were low, that is, very rare (e.g., “I will never be satisfied until I get all that I deserve,” proportion endorsed was 0.21, i.e., 21% of the participants agreed with this item).

In the third step, the first two authors compared, and contrasted interviews based on the ratings of the leader’s narcissism in order to specify the leadership behaviors for three groups of leaders with low, medium, and high levels of narcissism. For leader narcissism, the team rating of leader narcissism from Study 1 was used for the 18 teams represented in study 2 (see methods section of Study 1 for details on the team rating of leader narcissism). The third step involved several rounds of individual work and joint discussion. Step 3 resulted in additional codes that complemented the deductive codes from Step 2 (see Table 3). Wherever possible, we adopted terminology consistent with prior literature on leadership, entrepreneurial behavior and narcissism.

**TABLE 3:** Coding categories for qualitative data analysis (Study 2).

Code	Definition
Denying leadership role	Do not refer to themselves as leaders, do not perceive themselves as a leader
Showing humility	See themselves as not better or worse than anybody else
Accommodating behaviors	Avoid making decisions, balancing, are fair, motivate others
Serving others	Put the team members’ needs before their own, take over tasks for the team
Showing confidence as a leader	Perceive themselves as good leaders, show confidence with regard to leadership responsibilities
Shaping teamwork	Stir and decide but with an openness to others’ opinion
Communicating Vision	Have a vision, explicate clear goals in order to achieve this vision
Sharing leadership	Have an awareness for the needs of others, are decisive and integrate everybody’s opinions
Claiming leadership role	Perceive themselves as exceptional leaders, are deeply convinced of their leadership ability
Devaluating others	Express that they were more capable and more special than others, that they deserve to be treated differently, are upset if they don’t get what they deserve
Showing off	Steal the limelight, seek for a stage to shine, manage their impression
Exploiting others	Exploit the team, regard followers as an ends to achieve own goals, communicate excessively high expectations of their team members
Dominating others	Are authoritative and give orders, do not listen to others’ suggestions and ideas, ignore/omit team members

*^1^Coding categories derived from inductive analysis (see Methods section).*

*^2^Coding categories derived from deductive analysis (see Methods section).*

In a fourth step, these codes were presented to and discussed with other experts in the field at several occasions (e.g., friendly reviews, research colloquium, AOM annual meetings), and their feedback was incorporated.

### Coding Procedure and Reliability Check

To finalize our coding scheme of leadership behaviors, we compared and integrated codes stemming from deductive development of codes (deductive codes) and inductive analysis (inductive codes). Two of the authors coded the interviews using the final codes by assigning codes to every text segment that described leadership behaviors. Based on a detailed analysis of the coding of three random interviews, the coding scheme was adapted and the interviews were recoded. Overall, 558 text segments were coded using the coding scheme depicted in Table 3. The interrater reliability was checked based on a subset of 133 text segments (Cohen’s *kappa* = 0.90).

### Results

The following results are based on six interviews about team leaders that were rated low on leader narcissism ranging from 0.06 to 0.11, fourteen interviews about team leaders rated with medium levels of narcissism ranging from 0.17 to 0.52, and five interviews about team leaders rated high in narcissism by their team members, score ranging from 0.63 to 0.83. We compared and contrasted coding frequencies for these three groups of team leaders by creating a code-by-level of narcissism matrix (see Table 4). Each column presents coding frequencies for one specific group of team leaders (low, medium, high narcissism) as well as overall coding frequencies. This was followed by a detailed qualitative investigation of leadership behaviors that were typical for team leaders with low, medium, and high levels of narcissism.

**TABLE 4:** Coding frequencies by level of narcissism (Study 2).

Code	Low	Medium	High	Overall
Denying leadership role	13	6	0	19
Showing humility	5	7	0	12
Accommodating behaviors	15	17	7	39
Serving others	23	18	0	41
Showing confidence as a leader	7	16	4	27
Shaping teamwork	4	20	4	28
Communicating vision	2	21	4	27
Sharing leadership	5	85	13	103
Claiming leadership role	0	7	19	26
Devaluating others	0	26	12	38
Showing off	0	12	24	36
Exploiting others	0	33	35	68
Dominating others	2	43	33	78

*Table displays absolute numbers.*

We present the coding frequencies (see Table 4) and the behaviors of leaders with low and high levels of narcissism, i.e., those that were associated with lower team performance in the previous study and then in contrast at the behaviors shown by leaders with medium levels of narcissism, i.e., those associated with good team performance in the previous study.

### Leader Behaviors Associated With Different Levels of Leader Narcissism

#### Leader Behaviors Associated With Low Levels of Narcissism

For leaders who had a *low* narcissism value, we most frequently found the category *serving others*, which meant these entrepreneurs put the team members’ needs before their own and, for example, took over tasks that were unattractive. Second most frequent were *accommodating behaviors* with a focus on creating a good and balanced atmosphere in the team and by refraining from making decisions were salient only for entrepreneurial leaders low in narcissism. This was followed in frequency by *denying the leadership role*, which meant that entrepreneurial leaders would not refer to themselves as leaders or even explicitly disidentify with this role. However, this was not due to a lack of confidence in their leadership abilities, since we frequently found *showing confidence as a leader* (see Table 4).

To describe the behaviors recalled by the leaders *low in narcissism*, we can summarize that they focused very much on creating a nice atmosphere in the team and on being there for others (e.g., “If one of the team members had a wish, [I tried to] realize it.” or “I brought food to every meeting and stuff, so they were just taken with something like that too, that I was trying to get the team together somehow”). They had a serving attitude to leading the team (e.g., “I then only distributed tasks that they want to do and can do. Then I did the rest”), were hard-working and happy to take over tasks that others did not want to do (e.g., “I took care of the tasks that were left over.”). These team leaders would not talk about themselves as team leaders (e.g., “[I saw myself] not really as a team leader.” Or “I was more like a link between the team members”). Team leaders explicitly stated that they did not wish to be superior to the team members (e.g., “I would rather lead from within the team and not stand above the team.”). Thus, those team leaders showed humility (e.g., “I’m the team leader, but I’m not supposed to be a superordinate person in any way”) and shared the decision-making with the whole team (e.g., “Questions like this: yes, will you be able to do it by then? Or: is this deadline okay for you?”). However, this was not because of a lack of confidence in their ability as team leaders (e.g., “I think I managed that quite well, that they all don’t feel like they’re working for someone, but with me.”). When shaping the teamwork, they did it in a friendly way (“I think I’ve always been nice, but I’m explicit”).

However, the above described leader behaviors were perceived as problematic by the team members. While team members appreciated that nobody took the center stage (e.g., “Nobody steals the limelight or wants more attention than the others.”), they also reported a lack of structure and complained that decisions took long and involved a lot of discussion (e.g., “At the meetings there was always a lot of chaos.”) Team members of entrepreneurial leaders with low levels of narcissism reported that they wished for more structure and decisiveness (e.g., “At the next meeting I wanted to have a plan because I cannot work like that.”).

#### Leadership Behaviors Associated With High Levels of Narcissism

For team leaders *high* in narcissism, we found three categories as the most frequent. Namely *exploiting others*, which means they saw their team members as a resource to reach their goals, *dominating others*, meaning they were taking important decisions and controlled discussions, thus not taking other ideas on board, and *showing off*, meaning they wanted to be the center of attention and bragged about their achievements (see Table 4).

Team leaders high in narcissism, in contrast to those with low levels of narcissism, treated others with dominance (“Whatever I told them they were going to do, no fuss”) and showed very authoritarian decision-making (e.g., “I told them clearly for the presentation, we do it like this and this.”). Highly narcissistic leaders did not invite or even ignored team members’ ideas (e.g., “I had most of the business plan done and then one of the guys was insisting hard on doing some changes, but I did not want to do those changes because of my convictions,” or “I didn’t include their ideas, but I don’t feel bad because they were really not as good.”). Team leaders high on narcissism tended to exploit others to reach their goals and saw the team members as a means to an end (e.g., “I wouldn’t hesitate to kick someone out if they screwed up.” or “Okay, that doesn’t work that way. It’s easy, different demands on the whole and that’s why I reduced the team then”) and were even manipulative (e.g., “You have to communicate to people that everyone makes an important contribution and that you are extremely responsive to everyone. Whether you really do it, it’s always on a different page.”). They dominated the team (e.g., “I think I was kind of tyrannical in a way”). The leader’s own vision was pushed on others without asking them, rather than having a shared vision in the team (e.g., “I clearly communicated my shared vision to the others.”).

Team leaders high on narcissism had very high expectations of others; when they did not get what they thought they deserved, they reacted emotionally and usually rejected the other person (e.g., “[The team member] pissed me off […] and I kept my distance.”). These leaders made comments that pointed to feeling superior to others (e.g., “I made a lot of […] plans, and my guys didn’t even understand the plans, purely from an intellectual level.”). They perceived themselves as exceptional leaders (e.g., “I started relatively early, so at the age of 15 (…) on the board of directors. I did a good job back then”), claimed the leadership role for themselves (e.g., “It’s pretty clear I’m the leader.” or “But I’ve led several times before, and I know that I don’t do it so badly and that I’ve already led teams to success there and that’s why it’s sometimes almost more pleasant to lead when you know you can do it”), and did not share the stage with other team members (e.g., “As I am the most communicative person of the team, I decided to do it [an important presentation] on my own.”).

Team members perceived their team leaders’ dominance and showing off behavior as problematic as this behavior impeded their ability to contribute to advancing the entrepreneurial venture (e.g., “But with someone who talks and talks, you never get a chance to say anything.”). This even led to suboptimal decisions (e.g., “He just didn’t listen to me at all. I don’t know, I found that a bit unfair, and well, then I just said: yes, I do it as you like it. In the end it turned out that we should have done it the way I said.”). Team members also complained that their team leaders saw only themselves and not the team (e.g., “He had a problem with seeing the team because he really likes being the center of attention.”) and did not motivate the team (e.g., “Strengthening the team is not what our team leader did, but rather when he motivated us, it was always about the project but not the team itself.”).

#### Leadership Behaviors Associated With Medium Levels of Narcissism

Team leaders who had a medium level of narcissism reported as the most frequent categories a combination of sharing leadership behaviors, that is, for example, the leader asking team members for their opinions and involving them in decision making. At the same time dominating others, referring to behaviors that are authoritative, and exploiting others, meaning to see others as a means to achieve their goals, were frequent. However, these behaviors were reported relatively less frequently than for highly narcissistic team leaders (Table 4).

Leaders with medium levels of narcissism shared leadership and included the team members (e.g., “We talked a lot about it, I said again and again what I expected from the project, how I found it and the result now, and I think right now in such a small team it is incredibly important to be open”). Decisions were taken together (e.g., “We sat down together and we decided all together” or “Decisions were usually taken together, I initiated it but we actually took them together.”). They reflected that team members had individual needs and took them into consideration (e.g., “We had different backgrounds and therefore all had different views and priorities.”) when approaching team members to empower them (e.g., “Now (I had to) steer it in the right direction, without you saying to her, it sucks how you did it or I’d rather do it myself, because then it’s at least good”). They appreciated the input and opinions of their team members and also being challenged by them (e.g., “Quite interesting that all the ideas I had were not immediately accepted but were questioned by him–I found that very good, because I think that is the only way to improve, and thus I put a lot of emphasis on his opinion”).

Those team leaders were “go-getters”; they took responsibility and focused on task achievement (e.g., “I took over organizing: When do we meet, what do we do?”). Their statements showed that they were convinced to be good at organizing and that this is one reason that they were chosen as the team leader (e.g., “I guess organizing is something I am good at.”). These team leaders speak of themselves as a leader and felt comfortable with this role (e.g., “I did a good job at this and had a lot of ideas for the project”). If needed they were also dominant in decision-making and took the actions necessary to move the team project forward (e.g., “I put pressure on them–things had to happened”). Resorting to exploiting or manipulating if necessary (e.g., “So a bit manipulative maybe, too, so that in the end I get what I want after all.”) and admitted to being “hard or chilly or something or arrogant in places”. Despite these behaviors, they reflected their own behavior (e.g., “I also feel that in most conversations I overpower people when I get to know them”) and why they may show certain behaviors (e.g., “But on the other hand my arrogance might be due to overwhelming demands, because I don’t feel well, then I usually react arrogantly because I just don’t know how to react”). Those leaders also critically assessed their ideas and opinions at times (e.g., “They (the team member) couldn’t understand what I wanted for a long time, what gives me the feedback that it’s not so clear, my idea.”).

From the team members perspective, the team leaders’ dominance was noticed and expressed in the interviews (e.g., “He was a dominant leader, I would say”) and some of the team members also mentioned situations where it was difficult to collaborate with their leader (e.g., “What was a bit difficult was that he already dominated that very much. I mean, he had most of the ideas, it was his idea, you noticed that too, he spoke the most”). At the same time, they also recognized that their perspective was taken into consideration (e.g., “And at that point, I had the feeling he could listen sometimes. He *can* listen and he *can* understand if it makes sense for him”) and their opinion was invited (e.g., “He also wanted to hear my opinion often, to hear it in depth”). Altogether, team members perceived their entrepreneurial leaders as constructive (e.g., “He was never authoritarian but also not anti-authoritarian”). They appreciated that their leaders were keeping an eye on the progress toward the goals (e.g., “She always had the overview of what were the next steps […]; there was no need to worry about that”). Furthermore, they seemed to highly appreciate the sharing leadership behaviors (e.g., “He gave everybody the opportunity to contribute.”).

### Discussion

Our findings pinpoint the importance of complex patterns of leadership behaviors instead of more simplified notions that focus on singular behaviors. Whereas leader with both medium and high exhibited dominant behaviors, it was only in combination with exploiting behaviors and ‘showing off’ that this led to detrimental effects for team performance. In combination with participative and empowering leadership behaviors of the moderate narcissists, dominance was even effective in increasing team performance.

The behavioral patterns we found can be interpreted based on current leadership theory: The leadership behaviors of low narcissists can be related to servant leadership, which explicitly replaces leaders’ self-focus with concern for followers’ needs, which as such will instill purpose in followers and motivate them (Liden et al., 2008; Van Dierendonck, 2011). In fact, in our study, team leaders with low narcissism focused on their team members, ensuring everybody was heard and included; they had a strong relationship focus, were humble, and stepped back for others–but this did not lead their teams to success. Instead, team members complained about the lack of structure and guidance. Given the uncertain and ever-changing nature of entrepreneurship (McMullen and Shepherd, 2006), teams will look for guidance and ask for strong leadership (Maccoby, 2000; Padilla et al., 2007; Campbell and Campbell, 2009). Hence, it seems that in the nascent entrepreneurial context, the focus on tasks at hand and moving the venture forward is more important than a strong relationship orientation.

In fact, the most successful entrepreneurial teams had leaders who showed a lot of confidence and who were ‘do-ers.’ Those leaders dominated others in decision-making and required the team to achieve their goals. Their strong focus on getting things done, even if done in a dominant way, were highly effective in rallying together and to jointly advance ideas. These behaviors were typical of leaders with moderate levels of narcissism but also of leaders high in narcissism. At the same time, leaders with moderate narcissism, also allowed the team members to participate, asked for their input and empowered all members of the team to contribute their share in achieving the team vision. This is consistent with earlier research that found that the possibility to participate in decision-making and contributing own ideas had a positive effect on team performance (Wagner, 1994; Kim et al., 2010). We conclude that moderate leader narcissism combines directive and participative behaviors in a way that is highly effective in leveraging team performance in an ill-structured situation such as new venture creation.

Allowing team members to participate was a factor that was lacking in teams that had highly narcissistic team leaders. These leaders focused on themselves, showing off and having things done their way; this hindered team members to participate, to point out flaws in the idea, and to contribute their expertise to advancing the new venture. In a phase where there are no structure or routines and ideas develop and need to be shaped, the self-focus of the leader seems to be particularly detrimental, resulting in lower team performance eventually. Instead of driving the joint venture forward, leaders high in narcissism showed behaviors that were mainly focusing on showing themselves in a positive light and promoting themselves.

**FIGURE 2:**
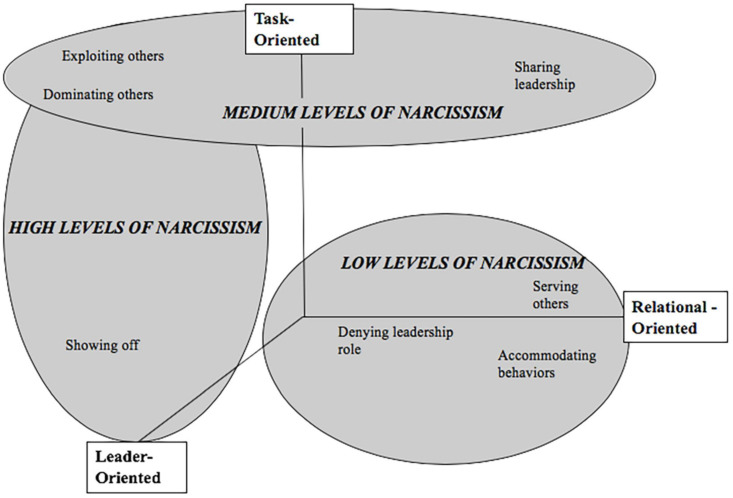
Patterns of leadership behaviors associated with high, medium, and low levels of narcissism (Study 2).

Figure 2 depicts the leadership behaviors and the patterns of behaviors that leaders of different levels of narcissism showed. Based on our findings, we argue that in categorizing the full spectrum of leader behaviors of narcissists, we need to consider *leader-orientation* in addition to task- and relationship-orientation, which has only very recently received attention (Schmid et al., 2019).

Whereas we investigated these leadership patterns in the compelling context of entrepreneurship, we assumed that these are not limited to the entrepreneurship context but can be generalized to different contexts.

## Study 3: An Examination of the Narcissistic Leadership Patterns Across Context

The aim of our research was to shed light on the narcissism-leadership-performance puzzle by investigating the two most promising avenues to understand it: *one*, it depends on the level of narcissism (Grijalva et al., 2015) and *two*, that it depends on the context whether narcissism in leaders in helping or hindering performance (Braun, 2017). Whereas Study 1 and 2 focused on the curvilinear relationship between leader narcissism and its underlying patterns of leadership behaviors in the entrepreneurship context, in Study 3, we focused on investigating the relevance of these patterns across context. In order to test if the perception of leaders showing the behavioral patterns we found in Study 2, we designed vignettes for the three patterns of behavior that correspond to leaders with low, medium and high levels of narcissism in two different contexts, i.e., entrepreneurship and the corporate context. In the vignettes, we describe a scenario where the participants were asked to imagine that they were working in a team in either a start-up (entrepreneurship context) or in a large international organization (corporate context) for a leader. We then looked at two types of leadership outcomes: Leader perceptions and perceptions of teamwork.

First, as the main aim of this paper is to understand leaders influence on performance, we examined on these leader-related outcomes: Satisfaction with the leader, perceived leader effectiveness, relationship quality, and LMX. In line with Grijalva et al. (2015), we assume that the behaviors associated with medium levels of narcissism will be seen as more satisfying and more effective than the patterns of behavior associated with high levels of narcissism. The Leader-Member-Exchange Theory (LMX) describes the relationship quality of leaders and their followers and predicts performance across contexts (Martin et al., 2016). We thus assume that the behavioral patterns associated with medium levels of narcissism show the better relationship quality and highest LMX.

Second, as we explored leader narcissism in a team context as called for by Braun (2017), we deem it important to investigate how the perception of the teamwork would be influenced by a leader showing the behavioral patterns of low, medium, or high narcissism. We thus examined team-related process variables: team trust/distrust, knowledge sharing and knowledge hiding, and team reflexivity. Trust in the team is an important team variable that has been shown to be influenced by leader behaviors. One the one hand, narcissistic leadership should undermine trustful relationships and increase distrust amongst team members because it includes manipulating followers and playing them off against each other (Peng et al., 2014; see also Knipfer and Schmid, 2019). In contrast, functional forms of leadership such as transformational or empowering leadership were found to increase trust and a positive team climate (Braun et al., 2013). We thus assume that leaders showing the patterns of behaviors associated with medium levels of narcissism relate to higher levels of trust and lower levels of distrust in the team in different contexts, whereas the patterns of behavior associated with high levels of narcissism will relate to lower levels of trust and higher levels of distrust in the team in different context.

Knowledge sharing and integrating the diverse expertise of team members is key for a team’s success. Recently, research has also looked at the dark side of knowledge sharing: Connelly et al. (2012, p. 65) described knowledge hiding as the “intentional attempt by an individual to withhold or conceal knowledge that has been requested by another person”. Drawing on evidence for the cascading effects of destructive leadership (e.g., Liu et al., 2017), we assume that knowledge hiding may be the result of role modeling and trickle-down effects, where highly narcissistic leaders foster team members’ knowledge hiding by showing self-interested and exploitative behaviors themselves. Moreover, leadership behaviors associated with high narcissism should be perceived as unfair treatment–and followers try to restore justice by deviant behaviors (e.g., Tepper et al., 2009). Indeed, Nevicka et al. (2011a) showed that team leaders’ narcissism was associated with lower team performance, and this effect was explained by the fact that narcissistic leaders inhibited information sharing among team members. Thus, we argue that the patterns of leader behaviors associated with high levels of narcissism should relate to more knowledge hiding intentions, while medium levels of narcissism should be related to less knowledge hiding intentions.

Adapting to new circumstances and learning from experience is another key process for team success (Knipfer et al., 2018; Otte et al., 2019; Santos and Neumeyer, 2021). Hence, we included team reflexivity, that is, the extent to which a team regularly discusses its goals and strategies to achieve its goals as a key team process (Schippers et al., 2007). Schippers et al. (2008) found that positive forms of leadership are associated with higher levels of team reflexivity. Leaders with moderate narcissism will communicate a clear and attractive visions of the joint endeavor, which in turn should facilitate reflection and discussion of strategies to achieve this vision together (Knipfer et al., 2018). In contrast, team members working with highly narcissistic leaders, who will manipulate them to pursue their own egoistic goals, will likely feel unsafe to speak up, point to mistakes, and openly discuss their experiences to learn from them.

Finally, as the aim of Study 3 is to understand whether the patterns of leadership identified in Study 2 apply across contexts, we argue that an additional important aspect to understand is how prototypical the leader behavior is perceived. Leader prototypicality was related to increase team cooperation (DeCremer et al., 2010). However, on the other hand, Van Knippenberg and Van Knippenberg (2005) found that sacrificing behaviors of leaders was related to leader effectiveness, and these effects were even stronger for less prototypical leaders. We thus explored the prototypicality of the three patterns of leader behavior in an entrepreneurship versus a corporate context.

### Sample and Procedure

We used a German panel provider to gather data from working adults from abroad range of occupations and industries. Two checks were employed to ensure data quality: (a) an ‘Honesty Check’: At the end of the questionnaire, we reminded participants that the data will be used for scientific purposes, and we asked them to indicate whether they answered the questions honestly and contentiously. We excluded participants who did fail the honesty check. Moreover, we excluded participants who finished the questionnaire under 3 min since we had to suspect that they did not answer the survey carefully enough. The final sample included *N* = 304 participants (151 females, 152 males, 1 diverse) with an average age of *M* = 46.87 (*SD* = 12.08) and a mean working experience of *M* = 24.65 (*SD* = 13.35).

We employed an 3 × 2 experimental design using the scenario technique (Aguinis and Bradley, 2014) to compare and contrast the effect of the leadership patterns that we had identified in Study 2. We varied the leadership behavior reflected in the scenario (i.e., the leadership behaviors that were associated with low, moderate, and high narcissism, Factor 1: leadership) and the context (a large corporate versus a start-up, Factor 2: context) to explore generalizability of our findings across contexts. Please see Appendix for the scenarios depicting the corporate context The scenario technique has been used successfully to explore the effects of leadership on follower outcomes before (e.g., Kovjanic et al., 2013). Six vignettes were purposefully designed to manipulate the perception of leadership behaviors. Participants were randomly assigned to one of the six experimental conditions; they were asked to read the scenario and to imagine that they were working in a team under the supervision of the described leader. Participants were then asked to rate their satisfaction with the leader, the leader’s effectiveness and the relationship quality with the leader. They were instructed to image that they have been working in a team, before we asked them to rate the level of trust and distrust toward the other team members and their intentions to hide knowledge from their peers. Finally, they were asked to rate the level of team reflexivity. Demographics were assessed at the end of the questionnaire.

### Manipulation Check

To examine whether the manipulation was successful, we used a selection of items that reflected different aspects of the manipulated leadership patterns, e.g., exploitative leadership (Schmid et al., 2019), autocratic leadership (DeLuque et al., 2008), empowering leadership (Zhang and Bartol, 2010), transformational leadership (Bass, 1999), and servant leadership (Pircher Verdorfer and Peus, 2014). Across contexts, the three leadership patterns were associated with significantly different evaluations (all *p* < 0.000) with the highly narcissistic leader being perceived as most exploitative and autocratic and least empowering, transformational, and servant. We found no significant differences for the factor context, nor any interaction effects of the two factors leadership and context. We conclude that the manipulation of the leadership patterns was successful and that the leadership patterns were similarly perceived across contexts as intended.

### Measures

We assessed satisfaction with the leader with three items from the Job Diagnostic Survey (Hackman and Oldham, 1974) that were also used by Judge (2000) to investigate leadership effects (α = 0.94), and leader effectiveness with four items of the German version of the MLQ (α = 0.94; Felfe, 2006). The relationship quality with the leader was measured using four items (α = 0.91; Schyns, 2006). LMX was measured using the German version of the LMX short scale by Graen and Uhl-Bien (1995), α = 0.92. Team reflexivity was assessed with five items (α = 0.90) by De Jong and Elfring (2010), team trust (α = 0.83) and distrust (α = = 0.76) were assessed by three items each based on Lowry et al. (2015), knowledge hiding and knowledge sharing were measured using six (α = 0.92) and three (α = 0.89) items (Connelly et al., 2012). Finally, we asked participants to rate the described leadership patterns in terms of leader typicality and leader familiarity using two items: “The described behaviors are typical for someone in a leadership position” and “The leadership behaviors are realistically described”.

### Results

In a first step, we examined whether the three different leadership patterns (reflecting low, moderate, and high narcissism) were related to leader-related outcomes using a multivariate analysis of variance: We found significant group differences in satisfaction with the leader, *F*(2,298) = 73.34, *p* < 0.000, leader effectiveness, *F*(2,298) = 103.28, *p* < 0.000, relationship quality, *F*(2,298) = 11.,27, *p* < 0.000, and LMX, *F*(2,298) = 80.94, *p* < 0.000. All *post hoc* tests were significant, p < 0.000, indicating that the three patterns of leadership differentially influenced all outcomes. Best outcomes were achieved for the leadership behaviors associated with low narcissism. Satisfaction with the leader, leader effectiveness, relationship quality, and LMX were rated better for the leadership pattern associated with *medium* narcissism compared to the leadership pattern associated with *high* narcissism. As expected, no differences were found for the factor context, nor did we find an interaction effect between the two experimental factors, leadership and context. This indicates that highly narcissistic leaders are perceived as less effective and less satisfying than moderate narcissists across contexts.

In a second step, we investigated group differences in regards to the team-related variables using multivariate analysis of variance again: We found significant group differences for the three leadership patterns (reflecting low, moderate, and high narcissism) with regards to team trust, *F*(2,298) = 7.22, *p* < 0.001, team reflexivity, *F*(2,298) = 10.54, *p* < 0.000, and knowledge sharing, *F*(2,298) = 3.58, *p* < 0.05. The mean values for these team process indicators were lowest for the leadership pattern associated with high narcissism and highest for the leadership patterns associated with low narcissism. For team distrust and knowledge hiding, we did not find significant group differences, nor did we find group differences for the factor context or any interaction effects of the two factors leadership and context. Inspection of the mean values for team trust indicated that, in the startup context, the leadership pattern associated with high narcissism was less harmful for team trust and distrust than in the corporate context.

### Exploratory Analyses

As Studies 1 and 2 suggested that the leadership pattern associated with moderate narcissism is functional and effective in a startup context, whereas the leadership pattern associated with high narcissism would be dysfunctional or even destructive, we performed two additional exploratory analyses, where we contrasted the leadership patterns associated with moderate versus high narcissism first in the startup context, and then in the corporate context. We found significant differences for satisfaction with the leader, leader effectiveness, relationship quality, and LMX for the startup context (all *p* < 0.000): As expected, the leadership pattern associated with moderate narcissism was shown to be more effective than the leadership pattern associated with high narcissism. No significant differences were found in regards to the team-related process indicators team trust, team distrust, team reflexivity, knowledge sharing, and knowledge hiding. However, in the corporate context, the results were different: Again, we found significant group differences for all leader-related outcomes, but we also found significant differences in terms of team trust, team distrust, and team reflexivity. The leadership pattern associated with moderate leader narcissism was related to higher team trust, less team distrust, and more team reflexivity compared to the pattern associated with high narcissism.

Second, we were interested whether the described leadership patterns were perceived as similarly typical and familiar across contexts. While the leadership behaviors that reflected low levels of narcissism were perceived as most effective by our participants, this leadership pattern was also perceived as significantly less realistic and less typical for a leader in both contexts compared to moderate and highly narcissistic leadership.

### Discussion

Overall, the results of our Study 3 point to the fact that the patterns of leader behaviors are valid across context. The context made no significant difference for any of the leader- or team-related outcome variables we investigated. Whereas this is in line with what we assumed, our data did not confirm that in fact the behavior patterns associate with a medium level of narcissism was always related to the most desirable outcomes. In fact, the behavioral patterns associated with low levels of narcissism were associated with highest levels of most leader-related and team-related outcomes, which is in line with the findings of Van Knippenberg and Van Knippenberg (2005) who found that sacrificing behaviors of leaders was related to leader effectiveness. However, our explorative analyses suggest that patterns associated with low levels of narcissism (i.e., serving and self-sacrificing behaviors) were perceived as least typical and least familiar, thus as behavior of a leader that is not as one would expect for both the entrepreneurship and the corporate context. Hence, while the low level of leader prototypicality seems to relate to positive perceptions, it is not a behavior that one would encounter often.

## General Discussion

In this paper, we set out to explore the narcissism-leadership-performance puzzle. Different explanations have been proposed for the puzzle that “research has not produced consensus concerning whether narcissistic leaders hinder or benefit their organizations” (Grijalva et al., 2015, p. 1). We took two most promising avenues, namely that the effects depend on the level of narcissism (Grijalva et al., 2015) and on the context (Braun, 2017), and opened the ‘black box’ of leadership behaviors associated with different levels of narcissism in the compelling context of entrepreneurship.

First, we investigated how leader narcissism relates to the performance of entrepreneurial teams. We contribute to what is according to Rosenthal and Pittinsky (2006) the most central question in research on leader narcissism, namely whether a narcissistic leader is beneficial or detrimental to performance. Whereas previous research has mainly sought to answer this question by examining a linear relationships of leader narcissism and its outcomes (Braun, 2017), we take a more nuanced look on this relationship. Our research expands previous findings and provides evidence for a *curvilinear* relationship between leader narcissism and entrepreneurial team performance. We also go beyond previous research that has focused on either individual follower outcomes or organizational-level outcomes, neglecting an intriguing setting to study narcissistic leadership, that is, *teams*, as also called for by Braun (2017) in her recent review of the literature. She concludes: While the scarce studies on narcissistic leaders of teams has pointed toward detrimental effects on the collaboration amongst the members of the team (e.g., Nevicka et al., 2011a), Wisse and Sleebos (2016) found that narcissistic leaders are not negatively perceived in the first place. Our findings further refine our understanding of the complex relationship between leader narcissism and team performance that goes beyond a ‘good versus bad’–perspective. Conclusively, the first important theoretical implication from our research is that narcissism unfolds the best outcomes in moderation in the entrepreneurship context.

Second, we further explored the curvilinear relationship between leader narcissism and entrepreneurial performance and open the black box to understand what actual leadership behaviors are underlying the curvilinear relationship that drive performance. We thereby answer the call for more in-depth qualitative explorations of leader narcissism by Braun (2017) and the call for investigations to uncover the different types of leader behaviors shown by narcissists (Grijalva et al., 2015). Our findings pinpoint the importance of complex behavioral patterns instead of more simplified notions that focus on singular behaviors. Our findings contribute to the state of the research on leadership in teams by pointing toward the significance of complex patterns of leadership behaviors of narcissists as proposed by the *behavior paradigm of leadership* (DeRue et al., 2011). Concretely, our results imply that the simplified view of “good” versus “bad” narcissistic leaders do not appropriately characterize the complex and multi-faceted nature of leadership behaviors that are associated with different levels of narcissism. Rather, our findings suggest that we need to consider the combination of distinct behaviors in examining the effects of leader narcissism. Whereas both medium and high narcissistic team leaders exhibited dominant behaviors, it was only in combination with exploiting behaviors and showing off that it led to detrimental effects on team performance. In combination with participative and empowering leadership behaviors of the moderate narcissists, dominance was even effective in increasing team performance. We are, to the best of our knowledge, the first to open the black box by exploring the behaviors that narcissistic leaders show, thereby allowing for theory elaboration on narcissistic leadership and its effects on team performance.

Third, the results of our multi-method and multi-source studies suggest that the most promising avenue to understand the narcissism-leadership-performance puzzle is that it depends on the levels of narcissism and more specifically that it depends on the patterns of behaviors narcissistic leaders show–the context seems to play a less important role. Study 3 provides first evidence that the leadership pattern associated with high levels of narcissism is detrimental for both, leader-related outcomes and team-related outcomes–and this across contexts. The leadership pattern associated with moderate levels of narcissism was related to more satisfaction with the leader and perceived leader effectiveness (see also Grijalva et al., 2015) as well as better relationship quality and LMX. At the same time, moderate narcissism seems to be conducive for team-related processes that have the potential to drive team performance, particularly in the corporate context: Here, it was associated with more trust and less distrust amongst team members as well as more team reflexivity, which is in line with findings by Schippers et al. (2008) and Braun et al. (2013) who also found beneficial effects of strong and visionary but participative and integrative leadership.

### Limitations and Future Research

While our study provides important contributions, its limitations need to be considered. Instead of using self-rating of leader narcissism, we decided to use follower ratings of leader narcissism as external ratings better reflect a behavioral style as proposed by Braun (2017). The team ratings of leader narcissism were obtained at the end of the venture creation program. As previous research has suggested that, while they may not be perceived as bad leaders at first, narcissism unfolds its negative outcomes only after some time (e.g., Braun, 2017), we think that our measure of leader narcissism was able to capture the impression of followers after several months of team work and collaboration with the team leader. Still, longitudinal designs would be promising to investigate further how the impact of leader narcissism on team performance unfolds over time.

Concretely, we measured team leader narcissism using a short version of the NPI, which is widely used (Podsakoff et al., 2003; Chang et al., 2010). It has been criticized for its potential multi-factorial nature mixing adaptive and maladaptive aspects of narcissism. But what was seen as critical proved to be valid to cover adaptive *and* maladaptive aspects of narcissism in our study. However, the calculation of a sum score over all items resulted in a low reliability in this study. Even though other researchers have found a similar level of reliability for the NPI narcissism score, future research should investigate the relationship of leader narcissism and team performance while accounting for the multi-faceted nature of narcissism as called for by Braun et al. (2018).

In Study 3, we adopted a scenario-based method, which allowed us to compare perceptions of the three patterns of leader behaviors across contexts. However, a limitation of this method is certainly that it is limited to perceptions and behavioral intentions and does not capture real reactions in a leader follower relationship. Future research should investigate the behavioral patterns in real-life settings to further validate our findings.

## Conclusion

Rosenthal and Pittinsky (2006, p. 619) stated that “the literature on narcissism in leadership is mainly devoted to answering one question: Is it good or bad for a leader to be a narcissist?” While previous research has produced conflicting findings when aiming to answer this question, our aim was to shed light on the narcissism-leadership-performance puzzle by investigating the two most promising avenues to understand it, namely that it depends on the level of narcissism (Grijalva et al., 2015) and on the context whether leader narcissism is helping or hindering team performance (Braun et al., 2018). Our studies show that a moderate level of leader narcissism was most beneficial for entrepreneurial team performance, and that highly narcissistic leaders can derail teams independent of the context. While leaders with moderate levels of narcissism show effective *and* destructive leader behaviors, it was not the behaviors *per se* but the complex combination of these behaviors that leveraged team performance eventually. Thus, it seems that the most promising avenue to understand the narcissism-leadership-performance puzzle is that it depends on the patterns of leadership behaviors associated with different levels of narcissism–and this across contexts.

## Data Availability Statement

The raw data supporting the conclusions of this article will be made available by the authors, without undue reservation.

## Ethics Statement

Ethical review and approval was not required for the study on human participants in accordance with the local legislation and institutional requirements. The patients/participants provided their written informed consent to participate in this study.

## Author Contributions

All authors have contributed to the design of the study and have participated jointly in collecting, analyzing and interpreting data, and in writing the manuscript. KK and ES collected, analyzed, and interpreted the data. ES and KK wrote the first draft of the manuscript. CP provided feedback for further development of the manuscript. All authors contributed to manuscript revision, read, and approved the submitted version.

## Conflict of Interest

The authors declare that the research was conducted in the absence of any commercial or financial relationships that could be construed as a potential conflict of interest.

## Appendix

### Scenario 1: Leadership Pattern of the Moderate Narcissist

You work in a large international company and have been part of a project team for a few months. You are working with four other colleagues and your manager on a very important project for a new customer.

Your manager expects you to work through the weekends and put your personal life on hold in favor of the project. The final presentation to the client is coming up soon and he keeps stressing that the presentation has to be perfect and he expects the whole team to give 200% to achieve his goal – which is to win this client.

While he is very authoritarian in his approach, has a tendency to give you instructions, and is often so convinced of his ideas that he won’t listen to any contrary opinions, there is also another side to him: as you prepare for the pitch, he cares a lot about your personal needs. You are very nervous before your first client presentation and he encourages and reinforces you a lot. He also makes sure to include everyone on what the optimal storyline might be, and makes sure everyone on the team agrees with what should be presented to the client.

### Scenario 2: Leadership Pattern of the High Narcissist

You work in a large international company and have been part of a project team for a few months. You are working with four other colleagues and your manager on a very important project for a new customer.

Your manager expects you to work through the weekends and put your personal life on hold in favor of the project. The final presentation to the client is coming up soon and he keeps stressing that the presentation has to be perfect and he expects the whole team to give 200% to achieve his goal – which is to win this client.

He is authoritarian in his approach, tends to give you orders and is often so convinced of his ideas that he does not listen to contrary opinions. As you prepared together for the final presentation, you realize that it is of central importance to him that the impression is created in the customer’s mind that he is the one behind the idea and that this alone is his success. He tells you clearly that he will make the presentation alone and that he is eagerly awaiting the moment when he can present himself on that stage. You and the team are not invited to the presentation.

### Scenario 3: Leadership Pattern of the Low Narcissist

You work in a large international company and have been part of a project team for a few months. You are working with four other colleagues and your manager on a very important project for a new customer.

Throughout the project, your supervisor always makes sure that you and the other team members are happy, and you feel that he is a true friend to everyone on the team. He does most of the work himself, asks you what part of the project you want to be involved in, and takes on the tasks that no one wants to do. The final presentation to the client is coming up soon.

As you prepare for the final presentation to the client, there is a lot of discussion among the team about how best to proceed and what the best storyline might be – of course, everyone has a different idea. Your supervisor always tries to make sure everyone is heard and listens to all points of view, but does not want to make a decision because it’s so important to him that everyone is happy with the outcome.
